# Exploring the interplay of discrimination perception, alexithymia, and shyness among hearing-impaired college students: a network approach

**DOI:** 10.3389/fpsyt.2026.1899912

**Published:** 2026-09-24

**Authors:** Jing Du, Delin Yu, Changsheng Hu

**Affiliations:** 1 School of Special Education, Fuzhou Polytechnic, Fuzhou, Fujian, China; 2 School of Psychology, Fujian Normal University, Fuzhou, Fujian, China; 3 School of Psychology, Northwest Normal University, Lanzhou, Gansu, China; 4 School of Teacher Education, Sichuan Vocational and Technical College, Suining, Sichuan, China

**Keywords:** alexithymia, discrimination perception, hearing-impaired college students, network analysis, shyness

## Abstract

**Background:**

Hearing**-**impaired college students face limitations in auditory and verbal communication, placing them at higher risk for mental health problems. While previous studies have mostly used traditional structural equation models to examine the relationship between discrimination perception and psychological adaptation issues, these approaches have difficulty capturing complex nonlinear associations and dynamic interactions between variables. This study adopted a network analysis perspective to explore the association patterns between discrimination perception, shyness, and alexithymia among hearing**-**impaired college students, and to identify influential intervention targets.

**Methods:**

This study was conducted in March 2026 on college students with hearing **-**impairments mainly from Fujian and Sichuan provinces using the Hearing-impaired Children perceived discrimination Questionnaire, the Social Withdrawal Subtypes and Peer Isolation Questionnaire, the Perth Alexithymia Questionnaire, and SPSS software for descriptive statistical analysis, and R software for network analysis.

**Results:**

The discrimination perception–shyness network had 12 cross-community edges (range: −0.044 to 0.057). Most were small (below 0.06), suggesting weak conditional associations. The strongest edge was Pd3–S5 (0.057). Pd3 showed the highest BEI (0.057). The discrimination perception–alexithymia network had 5 cross-community edges (range: 0.012 to 0.050). All were small (below 0.06). The strongest edge was Pd5–A2 (0.050). Pd6 showed the highest BEI (0.071).

**Conclusion:**

This study explored the relationships between discrimination perception, shyness, and alexithymia in hearing-impaired college students using network analysis methods. The Pd3 (Excluded from games) in the shyness network and Pd6 (Dishonesty) in the alexithymia network were identified as the highest bridge centrality nodes, suggesting that these are core symptoms connecting external stress to internal psychological adaptation difficulties and key triggers for social avoidance and emotional recognition difficulties in hearing-impaired college students. Thus, they represent the optimal targets for psychological intervention.

## Introduction

The hearing-impaired population constitutes a significant proportion of people with disabilities in China, accounting for approximately one quarter of the total disabled population (1). Hearing-impaired college students, as the primary focus of higher special education, face restricted auditory and verbal communication, which places them at elevated risk for mental health problems. They experience long-term challenges including insufficient social support, interpersonal difficulties, and social prejudice and discrimination. These stressors lead to a range of adaptation problems such as learning difficulties, significant mood fluctuations, social avoidance, and barriers to social integration. These conditions severely impair their mental health and development (2, 3).

Discrimination perception is an individual’s subjective experience of receiving unfair negative treatment directed at oneself or one’s group (4). Although the state has promoted an inclusive and equal social environment for people with and without disabilities through legal protections, policy support, and public campaigns, the stereotype that disability equates to incompetence persists (5). Moreover, when hearing-impaired college students encounter difficulties and setbacks, they tend to internalize negative societal labels, which may include assumptions of aloofness or withdrawal, as part of their self-evaluation, leading to self-stigma and devaluation of their social identity (6). According to modified labeling theory, when individuals endorse and internalize external negative labels, they experience self-depreciation and maladjustment, which in turn reinforces discrimination perception and ultimately creates a vicious cycle of negative cognition and psychological distress (7). For example, Pascual-Sanchez et al. (8) found that discrimination perception leads individuals to experience self-doubt and psychological distress, which reduces self-efficacy and generates self-shame, resulting in social withdrawal. Repeated experiences of discrimination continuously weaken an individual’s self-esteem and self-efficacy (9). Negative self-evaluation reduces an individual’s willingness to integrate into society, which decreases social participation (10) and leads to intense shyness. In summary, based on modified labeling theory, discrimination perception among hearing-impaired college students leads to social withdrawal and subsequently to shyness.

Emotion construction theory proposes that the brain interprets internal and external sensory cues to construct different emotional categories. However, when individuals experience long-term discrimination, the predictability of sensory information in specific situations decreases notably, which leads to alexithymia (11). For example, Savoca et al. (12) found a positive association between levels of discrimination perception in daily life and alexithymia, and identified alexithymia as a potential target for anxiety intervention in groups experiencing discrimination. In addition, Bibikar et al. (13) showed that in healthcare settings, anticipated discrimination perception among patients with chronic obstructive pulmonary disease (COPD) correlated positively with alexithymia, and alexithymia reduced patients’ satisfaction with medical care. In summary, based on emotion construction theory, discrimination perception among hearing-impaired college students leads to alexithymia.

Previous studies have primarily utilized traditional structural equation modeling (SEM), treating discrimination perception, shyness, and alexithymia as unitary constructs. These studies established mediation and moderation models to understand the relationships among these variables (8, 12). Moreover, SEM relies on assumptions of linearity and postulates latent variables to account for covariances among observed variables. This approach can be influenced by researcher-specified models and often struggles to capture complex nonlinear associations or dynamic interactions within a system (14).

Network analysis offers a novel approach to understanding complex psychological and behavioral systems by representing and analyzing system characteristics through network structures (14–16). By depicting interactions within a system as a network of “nodes” and “edges, “ this method allows for the identification of node centrality and inter-node relationships. This approach facilitates the identification of the most influential variables within the network and prepares the ground for both exploratory/practice-oriented and theory-driven research (17). Compared to traditional SEM, this method provides three key advantages. First, it avoids strict linear assumptions, effectively capturing nonlinear dependencies like feedback loops that SEM often struggles with (14). Second, a prominent feature of network analysis is its visualization capacity. It offers a clear visual representation of complex dynamics, illustrating nodes and their connections (14, 18). By employing partial correlations, network analysis controls for the influence of other nodes, providing a more accurate depiction of the true relationships between variables (19). Importantly, network analysis can effectively explore the structure of high-dimensional data even in the absence of prior theoretical knowledge regarding variable associations (14). Third, its holistic perspective incorporates all observed variables into a unified network framework. This approach overcomes the limitations of structural equation modeling, which often struggles to unravel the complex dynamic relationships between variables and to capture the interactive processes within the system (14, 20). Its strength lies in revealing complex interactions and identifying key nodes with the greatest systemic impact, providing more precise targets for interventions.

Therefore, this study employs network analysis to investigate the relationships among discrimination perception, shyness, and alexithymia in hearing-impaired college students. Compared to traditional SEM, this approach does not rely on linear assumptions and can identify potential intervention targets, providing empirical support for alleviating the negative effects of discrimination perception and developing targeted intervention strategies.

## Methods

### Participants

In March 2026, a stratified cluster random sampling method was adopted. Using hearing-impaired student classes as sampling clusters, eight universities offering programs for hearing-impaired students—including Tianjin University of Technology’s Deaf College and Fuzhou Vocational and Technical College—were selected as sampling units. A total of 500 questionnaires were distributed and collected by college counselors at each institution. Inclusion criteria were: confirmed hearing impairment through audiological examination, ability to complete the questionnaire independently, and voluntary participation. Exclusion criteria were: intellectual or mental disorders, or invalid responses such as patterned answering or extensive missing data. Invalid responses were identified by the following criteria: completion time less than 3 minutes, more than 10 consecutive identical responses, missing rate exceeding 33.3%, and duplicate or logically inconsistent responses. After excluding 37 invalid questionnaires, 463 valid responses were retained, yielding a valid response rate of 92.60%. The sample comprised 208 males and 255 females, aged 18 to 22 years (M = 20.49, SD = 1.33). Basic information including hearing loss degree, age of hearing loss onset, and parental hearing status was also collected. For the remaining sparse missing values in valid questionnaires, series mean imputation was applied. Prior to completing the questionnaire, research staff provided a detailed explanation of the guidelines and precautions. This study was approved by the Ethics Committee of Sichuan Vocational and Technical College (Approval No: SCSXLXH202502003-2) and complied with the provisions of the Declaration of Helsinki. Written informed consent was obtained from all participants prior to data collection. The informed consent procedure was as follows: (1) Before questionnaire administration, each participant received a paper-based informed consent form detailing the research purpose, procedures and duration (approximately 10–15 minutes), the voluntary nature of participation, the right to withdraw at any time without reason or penalty, anonymization and confidential data storage, potential benefits (gaining self-awareness of one’s psychological status and receiving popular psychology books as gifts), potential risks (transient negative emotions triggered by recalling discriminatory experiences), and the corresponding psychological support arrangements; (2) After fully understanding all content, participants voluntarily signed and dated the informed consent form; for participants under 18 years of age, additional written guardian consent was obtained.

### Measures

#### Hearing-impaired children perceived discrimination questionnaire

This study used the Hearing-impaired Children Perceived Discrimination Questionnaire developed by Xue (21) to measure discrimination perception among hearing-impaired college students. Although the scale was originally developed for children, all items are designed around universal discriminatory experiences in campus, community, and public settings—such as “interactions with classmates at school, ” “being stared at differently by neighbors, ” and “external rejection and negative evaluations”—which closely align with the real-life discriminatory experiences of 18–22 year old hearing-impaired college students, supporting its content appropriateness for this population. The questionnaire contains 7 items rated on a 4-point Likert scale (e.g., “I think most people do not like me”), with higher total scores indicating more severe discrimination perception. In this study, the Cronbach’s α coefficient was 0.877. Confirmatory factor analysis indicated that the single-factor model fit the data well: χ² = 41.661, df = 14, χ²/df = 2.976, RMSEA = 0.065, CFI = 0.979, TLI = 0.969, SRMR = 0.028. Confirmatory factor analysis indicated that the single-factor model fit the data well: χ² = 41.661, df = 14, χ²/df = 2.976, RMSEA = 0.065, CFI = 0.979, TLI = 0.969, SRMR = 0.028.

#### Social withdrawal subtypes and peer isolation questionnaire

This study used the shyness subscale of the Social Withdrawal Subtypes and Peer Isolation Questionnaire, developed by Coplan (22) and revised by Nelson (23). This subscale contains 6 items rated on a 5-point Likert scale (e.g., “I feel tense in social situations”). Higher total scores for subjects indicate more severe shyness. In this study, the Cronbach’s alpha coefficient for the shyness dimension was 0.855.

#### Perth alexithymia questionnaire

This study used the Perth Alexithymia Questionnaire developed by Preece (24) and revised by Niu (25). The questionnaire includes four dimensions: Negative - Difficulty Identifying Feelings, Positive - Difficulty Identifying Feelings, Negative - Difficulty Describing Feelings, and Positive - Difficulty Describing Feelings. The questionnaire uses a 7-point Likert scale (e.g., “I tend to ignore how I feel.”). Higher total scores for subjects indicate more severe alexithymia. In this study, the Cronbach’s alpha coefficient for the total scale was 0.944, with Negative - Difficulty Identifying Feelings at 0.820, Positive - Difficulty Identifying Feelings at 0.827, Negative - Difficulty Describing Feelings at 0.813, and Positive - Difficulty Describing Feelings at 0.791.

### Statistical analysis

SPSS 26.0 was used to calculate the means and standard deviations of network nodes. The network model was constructed using R version 4.5.0 (key packages: qgraph 1.9.8, bootnet 1.5, networktools 1.5.2, psych 2.4.3, and mice 3.16.0), employing a Gaussian graphical model to fit the data (16). As an undirected network, the edges of the Gaussian graphical model represent partial correlations between two nodes after controlling for all other nodes in the network (16). Combining the graphical “least absolute shrinkage and selection operator”(LASSO) algorithm and the “extended Bayesian information criterion” (EBIC) for model selection yields a more stable and more interpretable network (26).

Spearman correlations were used as input to the Gaussian graphical model, the EBIC hyperparameter γ was set to 0.5, and the network was visualized using the Fruchterman-Reingold algorithm. The hyperparameter γ controls the penalty on model complexity (Foygel & Drton, 2010), with larger values yielding sparser networks. Setting γ to 0.5 is the most widely adopted default in psychological network analysis (Epskamp & Fried, 2018), and simulation studies have confirmed that, under moderate sample sizes, this value provides an optimal and robust balance between sensitivity (detecting true edges) and specificity (controlling false edges) (Foygel & Drton, 2010; Epskamp et al., 2018).

Two distinct networks were constructed, namely, the Discrimination Perception and Shyness network, and the Discrimination Perception and Alexithymia network. The layout was determined using the Fruchterma –Reingold algorithm.

The evaluation of node bridge centrality was performed using the R package networktools (27). The bridge centrality metric selected for this study was Bridge Expected Influence (BEI), which represents the sum of a node’s edge weights with nodes in other communities. This metric is particularly suitable for networks with both negative and positive associations, where a higher BEI indicates a greater degree of association with other communities (27).

Finally, network robustness was tested using the R package bootnet (15, 16). The stability of the BEI was assessed using case-dropping bootstrapping (1, 000 samples) and the correlation stability coefficient (CS-coefficient), which ideally should be greater than 0.5. Edge accuracy tests, edge difference tests, and bridge centrality difference tests were also conducted.

## Results

### Descriptive statistics and correlation analysis

**Tables 1** and **2** display the means, standard deviations, and BEI values for the variables in the Discrimination Perception-Shyness network and the Discrimination Perception-Alexithymia network, respectively. **Table 3** presents the correlation analyses among the variables.

**Table 1:** Means, standard deviations, and BEI for variables in the discrimination perception-shyness network (n = 463).

Discrimination perception	Mean ± SD	BEI
Pd1. Lack of communication	2.02 ± 0.926	0.028
Pd2. Not being liked	1.90 ± 0.929	0.053
Pd3. Excluded from games	1.92 ± 0.916	0.057
Pd4. Being nicknamed	1.87 ± 0.951	0.015
Pd5. Treated differently	1.95 ± 0.993	0.007
Pd6. Dishonesty	1.70 ± 0.883	0.027
Pd7. Being scolded or hit	1.74 ± 0.957	-0.005

Shyness	Mean ± SD	BEI
S1. Feeling very shy	3.25 ± 0.946	-0.04
S2. Feeling nervous when going out	3.27 ± 0.967	0.121
S3. Feeling nervous in interactions	3.19 ± 1.039	0.000
S4. Feeling nervous in social situations	3.31 ± 0.988	0.010
S5. Avoiding social contact	2.74 ± 1.114	0.092
S6. Feeling nervous at gatherings	3.26 ± 0.991	0.003

**Table 2:** Means, standard deviations, and BEI for variables in the discrimination perception-alexithymia network (n = 463).

Discrimination perception	Mean ± SD	BEI
Pd1. Lack of communication	2.02 ± 0.926	0.000
Pd2. Not being liked	1.90 ± 0.929	0.024
Pd3. Excluded from games	1.92 ± 0.916	0.012
Pd4. Being nicknamed	1.87 ± 0.951	0
Pd5. Treated differently	1.95 ± 0.993	0.050
Pd6. Dishonesty	1.70 ± 0.883	0.071
Pd7. Being scolded or hit	1.74 ± 0.957	0

Alexithymia dimension	Mean ± SD	BEI
A1. Negative - Difficulty Identifying Feelings	16.50 ± 3.352	0.057
A2. Positive - Difficulty Identifying Feelings	16.46 ± 3.046	0.101
A3. Negative - Difficulty Describing Feelings	16.369 ± 3.338	0
A4. Positive - Difficulty Describing Feelings	16.21 ± 3.274	0

**Table 3:** Correlation analysis of the data (n = 463).

Variable	M	SD	Correlation		
			1	2	3
1. discrimination perception	13.092	4.975	1		
2. Alexithymia	65.531	12.331	0.268**	1	
3. Shyness	19.015	4.611	0.188**	0.335**	1

**p < 0.01. M, Mean; SD, Standard Deviation. 1 = Discrimination perception. 2 = Alexithymia. 3 = Shyness.

### Common method bias

To test for common method bias, the Harman single-factor test was conducted. Four factors had eigenvalues > 1, and the first factor explained 34.17% of the variance, which is below the 40% threshold. This indicates that no significant common method bias is present (28). A confirmatory factor analysis was conducted to test for common method bias by specifying a single latent factor onto which all self-report items were loaded. The model fit the data poorly, χ²/df = 9.344, CFI = 0.592, TLI = 0.561, RMSEA = 0.134, SRMR = 0.135, suggesting that a single common method factor does not adequately account for the observed covariances among the items (28).

### Network analysis

#### Network structure

The network structure of discrimination perception and shyness among hearing-impaired college students (**Figure 1A**) showed that there were 12 associations paths across discrimination perception and shyness, of which 10 were positively correlated and 2 were negatively correlated, with edge weights ranging from -0.044 to 0.057. Pd1 (lack of communication) was positively correlated with two nodes of shyness, with the strongest positive correlation being S2 (feeling nervous when going out), with an edge weight of 0.025. Pd2 (not being liked) was positively correlated with two shyness nodes, with the strongest positive correlation being S2 (feeling nervous when going out), with an edge weight of 0.046. Pd3 (excluded from games) was positively correlated with S5 (avoiding social contact), with an edge weight of 0.057. Pd4 (being nicknamed) was positively correlated with S2 (feeling nervous when going out), with an edge weight of 0.015. Pd5 (treated differently) was positively correlated with S4 (feeling nervous in social situations), with an edge weight of 0.007. Pd6 (dishonesty) was positively correlated with three shyness nodes, with the strongest positive correlation being S2 (feeling nervous when going out), with an edge weight of 0.037, and was negatively correlated with S1 (feeling very shy), with an edge weight of -0.044. Pd7 (being scolded or hit) was negatively correlated with S4 (feeling nervous in social situations), with an edge weight of -0.005.

**Figure 1:**
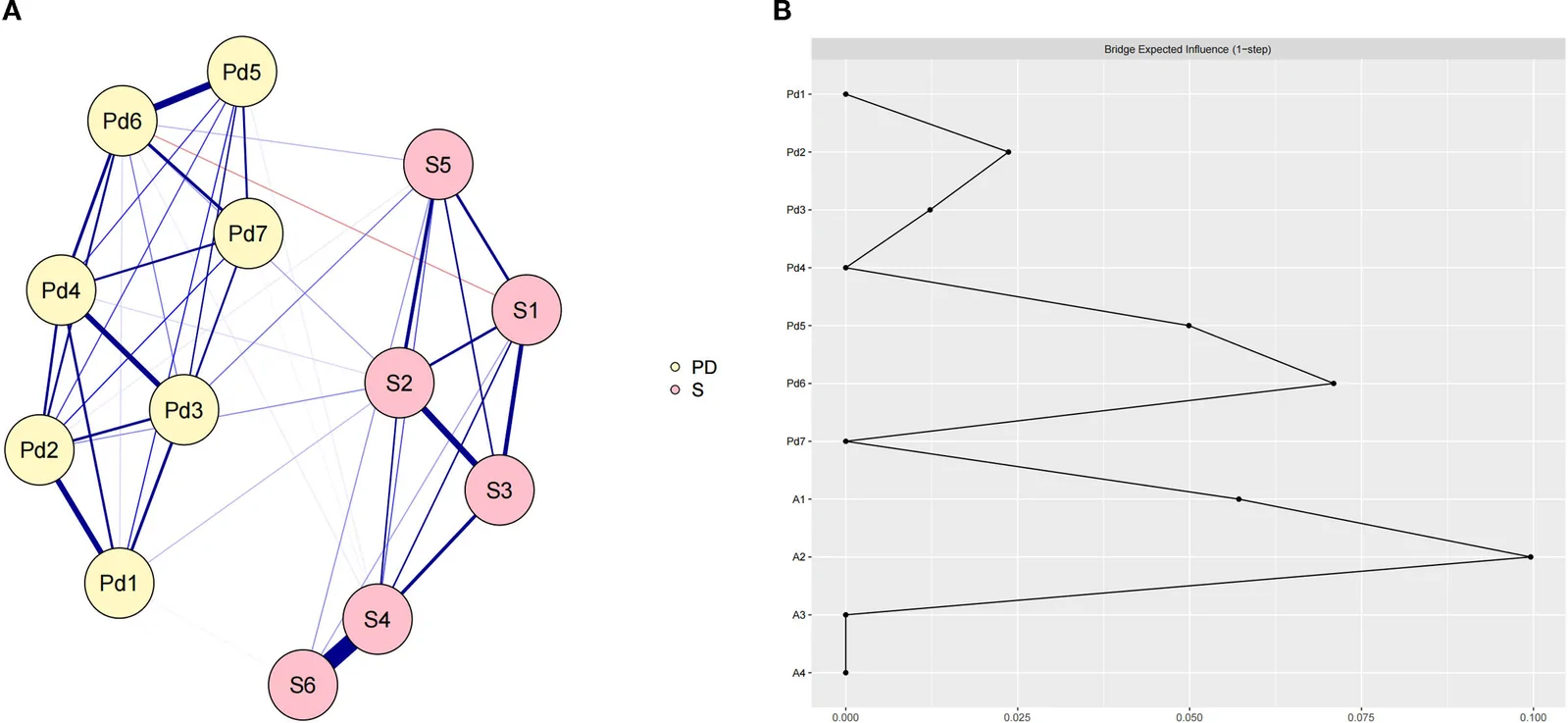
**(A)** Network of discrimination perception and shyness among hearing-impaired college students. discrimination perception: Pd1 (lack of communication), Pd2 (not being liked), Pd3 (excluded from games), Pd4 (being nicknamed), Pd5 (treated differently), Pd6 (dishonesty), Pd7 (being scolded or hit). Shyness: S1 (feeling very shy), S2 (feeling nervous when going out), S3 (feeling nervous in interactions), S4 (feeling nervous in social situations), S5 (avoiding social contact), S6 (feeling nervous at gatherings). Blue lines represent positive associations; red lines represent negative associations. Thicker and more saturated lines indicate stronger associations. See **Supplementary Appendix 11** for details. **(B)** Bridge expected influence values for nodes in the discrimination perception and shyness network among hearing-impaired college students. discrimination perception: Pd1 (lack of communication), Pd2 (not being liked), Pd3 (excluded from games), Pd4 (being nicknamed), Pd5 (treated differently), Pd6 (dishonesty), Pd7 (being scolded or hit). Shyness: S1 (feeling very shy), S2 (feeling nervous when going out), S3 (feeling nervous in interactions), S4 (feeling nervous in social situations), S5 (avoiding social contact), S6 (feeling nervous at gatherings). See **Supplementary Appendix 12** for details.

The associations within the shyness community were all positive, with the strongest association between S6 (feeling nervous at gatherings) and S4 (feeling nervous in social situations), with an edge weight of 0.712.

The associations within the discrimination perception community were all positive, with the strongest association between Pd5 (treated differently) and Pd6 (dishonesty), with an edge weight of 0.317. The specific values of all edge weights in the network are shown in **Supplementary Appendix 1**.

The network structure of discrimination perception and alexithymia among hearing-impaired college students (**Figure 2A**) showed that there were 5 association paths across discrimination perception and alexithymia, all of which were positively correlated, with edge weights ranging from 0.012 to 0.050. Pd2 (not being liked) was positively correlated with A2 (Positive - Difficulty Identifying Feelings), with an edge weight of 0.024. Pd3 (excluded from games) was positively correlated with A1 (Negative - Difficulty Identifying Feelings), with an edge weight of 0.012. Pd5 (treated differently) was positively correlated with A2 (Positive - Difficulty Identifying Feelings), with an edge weight of 0.050. Pd6 (dishonesty) was positively correlated with two alexithymia nodes, with the strongest positive correlation being A1 (Negative - Difficulty Identifying Feelings), with an edge weight of 0.045.

**Figure 2:**
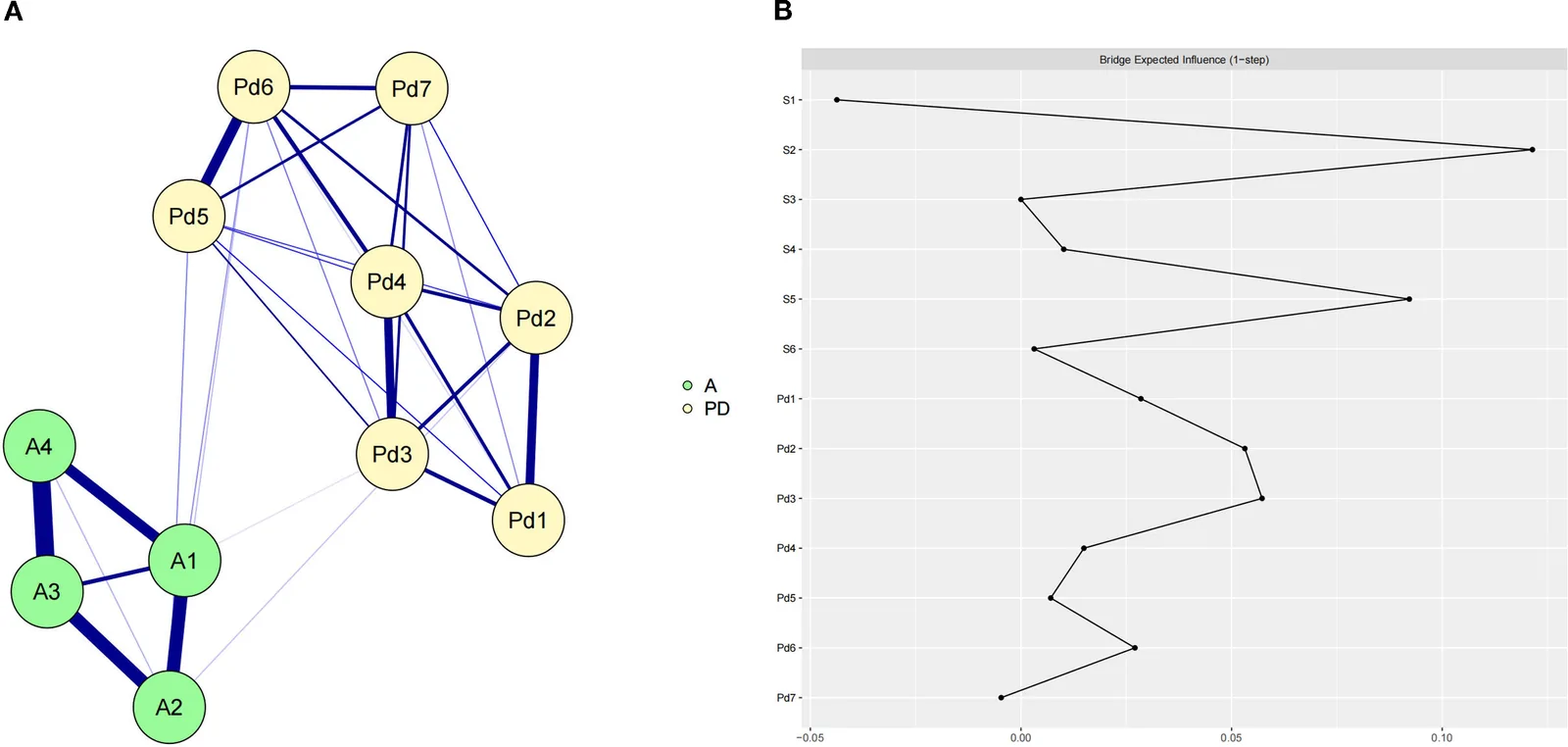
**(A)** Network of discrimination perception and alexithymia among hearing-impaired college students. discrimination perception: Pd1 (lack of communication), Pd2 (not being liked), Pd3 (excluded from games), Pd4 (being nicknamed), Pd5 (treated differently), Pd6 (dishonesty), Pd7 (being scolded or hit). Alexithymia: A1 (Negative - Difficulty Identifying Feelings), A2 (Positive - Difficulty Identifying Feelings), A3 (Negative - Difficulty Describing Feelings), A4 (Positive - Difficulty Describing Feelings). Blue lines represent positive associations; red lines represent negative associations. Thicker and more saturated lines indicate stronger associations. See **Supplementary Appendix 13** for details. **(B)** Bridge expected influence values for nodes in the discrimination perception and alexithymia network among hearing-impaired college students. discrimination perception: Pd1 (lack of communication), Pd2 (not being liked), Pd3 (excluded from games), Pd4 (being nicknamed), Pd5 (treated differently), Pd6 (dishonesty), Pd7 (being scolded or hit). Alexithymia: A1 (Negative - Difficulty Identifying Feelings), A2 (Positive - Difficulty Identifying Feelings), A3 (Negative - Difficulty Describing Feelings), A4 (Positive - Difficulty Describing Feelings). See **Supplementary Appendix 14** for details.

The associations within the alexithymia community were all positive, with the strongest association between A3 (Negative - Difficulty Describing Feelings) and A4 (Positive - Difficulty Describing Feelings), with an edge weight of 0.471.

The associations within the discrimination perception community were all positive, with the strongest association between Pd5 (treated differently) and Pd6 (dishonesty), with an edge weight of 0.307. The specific values of all edge weights in the network are shown in **Supplementary Appendix 1**.

#### The bridge expected influences

The BEI results in the discrimination perception and shyness network for hearing-impaired college students (**Figure 1B**) showed that Pd3 (excluded from games) had the highest bridge expected influence in the discrimination perception community (BEI = 0.057, 95%CI 0.024-0.130), followed by Pd2 (not being liked, BEI = 0.053, 95%CI 0.017-0.112). These nodes were positively correlated with a wider range of shyness symptoms, suggesting that they may be associated with shyness in hearing-impaired college students. The correlation stability coefficient (CS-coefficient) for BEI in this network was 0.283. Although this value exceeded the minimum recommended threshold of 0.25, it fell below the preferred threshold of 0.50, indicating that the bridge centrality estimates had limited stability and should be interpreted as preliminary findings (see **Supplementary Appendix 3**). The 95% bootstrap confidence intervals for edge weights ranged from small to moderate (see **Supplementary Appendix 4**), suggesting limited to moderate accuracy of the edge estimates. Edge weight difference tests and BEI difference tests were reported in **Supplementary Appendices 5** and **6**.

The BEI results in the discrimination perception and alexithymia network for hearing-impaired college students (**Figure 2B**) showed that Pd6 (dishonesty) had the highest bridge expected influence in the discrimination perception community (BEI = 0.071, 95%CI 0.027-0.156), followed by Pd5 (treated differently, BEI = 0.050, 95%CI 0.019-0.110). These nodes were positively correlated with a wider range of alexithymia symptoms, suggesting that they may be associated with alexithymia in hearing-impaired college students. The correlation stability coefficient (CS-coefficient) for BEI in this network was 0.361. Although this value exceeded the minimum recommended threshold of 0.25, it fell below the preferred threshold of 0.50, indicating that the bridge centrality estimates had limited stability and should be interpreted as preliminary findings (see **Supplementary Appendix 7**). The 95% bootstrap confidence intervals for edge weights ranged from small to moderate (see **Supplementary Appendix 8**), suggesting limited to moderate accuracy of the edge estimates. Edge weight difference tests and BEI difference tests were reported in **Supplementary Appendices 9** and **10**.

## Discussion

Network analysis revealed distinct patterns of associations between discrimination perception, shyness, and alexithymia in hearing-impaired college students.

First, the strongest positive association was observed between node Pd2 (not being liked) and S5 (avoiding social contact), suggesting that ‘not being liked’ may contribute to shyness. For example, Schoch et al. (29) found through two situational tests and one field experiment that social exclusion in the form of not being liked significantly influenced individuals’ social avoidance and attribution styles. Second, the strongest positive association was found between node Pd5 (treated differently) and A2 (Positive Difficulty Identifying Feelings), suggesting that ‘being treated differently’ is strongly associated with the ‘Positive - Difficulty Identifying Feelings’ dimension of alexithymia. For instance, Hamel et al. (30) reported that experiences of mistreatment, such as abuse, significantly increased the risk of alexithymia in adolescents. Specifically, more frequent abuse experiences were related to a poorer ability to identify and express emotions, including positive emotions, in adolescents.

However, this study revealed a negative association between Pd6 (dishonesty) and S1 (shyness), a counterintuitive finding that warrants further investigation. First, Talwar et al. (31) reported that sibling conflict and interaction significantly promote the development of lying skills in children. In contrast, shy children, often lacking such opportunities for social interaction, tend to exhibit weaker deceptive abilities. Specifically, shy children often have smaller social circles and lack environments that allow them to practice and refine lying skills through complex interpersonal interactions, resulting in a lower frequency of dishonest behavior. Second, Talwar et al. (31) also reported that an authoritative parenting style and a lack of positive family emotions encourage children to tell prosocial lies for the sake of politeness. Specifically, shy children tend to show greater compliance. In order to avoid negative evaluation and conflict, they often strictly adhere to social rules, including honesty, thereby exhibiting a lower tendency toward dishonesty.

The bridge expected influence (BEI) of discrimination perception on nodes in the shyness and alexithymia networks among hearing-impaired college students may reflect conditional associations between discrimination perception and shyness (32) as well as alexithymia (33). Within the network structure linking discrimination perception to shyness, the relatively high BEI of the bridge node Pd3 (excluded from games) highlights the peer activity exclusion challenges faced by hearing-impaired college students. They may experience greater difficulty participating in peer recreational activities compared to their hearing peers, which may be associated with various interpersonal withdrawal and shy behaviors. A notable pattern exists between this exclusion and withdrawal. For example, Buhs & Ladd (2001) found that rejected children are more likely to experience negative peer treatment including exclusion from peer activities and refusal of group entry bids, which is associated with increased loneliness and school avoidance (34). This negative interpersonal experience of being excluded from games may be associated with increased internalizing symptoms such as anxiety and depression, potentially contributing to further social avoidance among hearing-impaired college students.

In the network structure linking discrimination perception to alexithymia, the relatively high BEI of the bridge node Pd6 (dishonesty) suggests a potential association: the interpersonal mistrust and negative evaluation reflected by perceived dishonesty may be linked to difficulties in emotional awareness among hearing-impaired college students. When individuals feel unfairly accused or mistrusted, they may become more guarded in expressing their emotions, potentially limiting their opportunities to practice emotional identification and description. Taylor et al. (1997) proposed that alexithymia develops partly through disrupted emotional socialization processes, where negative interpersonal experiences interfere with the learning and labeling of emotional states (33). Consistent with this, Eisenberger et al. (2003) demonstrated that experiences of social exclusion are associated with difficulties in emotion regulation, providing a relevant framework for understanding the relationship between discriminatory experiences and emotion recognition difficulties (35). Our network findings align with these perspectives by identifying perceived dishonesty as a discrimination experience that may be conditionally associated with alexithymia features in this population.

Theoretically, our findings offer preliminary support for extending modified labeling theory (7). This theory originally posits that internalizing external negative labels leads to self-devaluation and social maladjustment. Using network analysis, we tentatively suggest that among hearing-impaired college students, this internalization process may not occur uniformly but rather through specific nodes such as “excluded from games” (Pd3) and “dishonesty” (Pd6), which show relatively stronger cross-community connectivity. The association between Pd3 and shyness-related symptoms suggests a potential pathway of “perceived exclusion in peer activities → social avoidance → internalizing symptoms, “ which may be associated with stable shyness traits. This exploratory finding extends modified labeling theory from a macro-level mechanism of social identity devaluation to a more nuanced, symptom-level process. Moreover, our results offer preliminary support for emotion construction theory Barrett, L. F. (2017). by identifying “dishonesty” (Pd6) as a discrimination-related node that may be associated with impaired emotional cognitive functioning (36). Previous studies have shown that chronic social exclusion and unfair treatment may reduce individuals’ ability to predict and recognize their own emotional cues (37); our study provides network-based exploratory evidence consistent with this perspective.

Practically, Pd3 and Pd6, due to their relatively higher bridge centrality in the respective networks, may warrant attention as potential points for future intervention research among hearing-impaired college students. For Pd3 (excluded from games), inclusive peer activity programs and social skills training in recreational contexts may be considered. Buhs & Ladd (2001) demonstrated that structured peer group interventions focusing on collaborative activities and shared experiences are associated with reduced social withdrawal and increased peer acceptance among socially isolated children (34). Extending such approaches to hearing-impaired college students may help rebuild positive peer interaction experiences in low-threat recreational scenarios. For Pd6 (dishonesty), interventions addressing interpersonal trust and emotional communication may be relevant. Research on emotion regulation by Gross (2002) and mindfulness training by Kabat-Zinn (2003) suggests that enhancing emotional awareness and non-judgmental attention may be helpful for improving emotion recognition difficulties (38, 39). Additionally, given that alexithymia involves difficulties in emotion recognition and expression, art-based therapy or emotion-focused counseling Pifalo, T. (2006) may help hearing-impaired students externalize emotions that are otherwise difficult to articulate, potentially reducing alexithymia-related difficulties (40).

In summary, from an exploratory bridge centrality perspective, Pd3 (excluded from games) and Pd6 (dishonesty) emerged as nodes with relatively higher cross-community connectivity in the present networks. These findings are preliminary and should not be interpreted as identifying definitive intervention targets. Given the cross-sectional design of this study, the relatively small cross-community edge weights (mostly below 0.06), and the BEI stability coefficients that did not reach the preferred threshold (0.283 and 0.361, below 0.50), these results are more suitable for generating future hypotheses rather than directly guiding intervention design. They may inform future research to help campus mental health services better understand discrimination-related psychological difficulties among hearing-impaired college students.

### Limitations and future research

This innovative study explored the network association patterns of discrimination perception with shyness and alexithymia in hearing-impaired college students. However, the study has several limitations. Based on the findings, we propose future research directions as detailed below.

First, limitations in study design and measurement. This study relied solely on self-report scales, which may be subject to social desirability bias. Furthermore, information on participants’ daily communication mode (sign language/oral/bilingual) and hearing aid or cochlear implant use was not collected. These factors have been shown in prior research to be closely associated with discrimination experiences and psychological adjustment in hearing-impaired populations, and their absence limits a comprehensive characterization of sample heterogeneity. Future research should incorporate these variables to more precisely examine the moderating role of hearing rehabilitation conditions in the association between discrimination perception and psychological adjustment. Additionally, this study employed a cross-sectional design, which precludes causal inference or examination of dynamic mechanisms. Subsequent studies could adopt longitudinal designs and construct cross-lagged panel mediation models to systematically examine temporal changes and directional relationships among the three variables. Further, the long-term effects of discrimination perception on social adjustment in hearing-impaired college students remain underexplored, and future research could extend this line of inquiry.

Second, methodological design limitations. This study used separate network models to examine the associations between discrimination perception and shyness, and between discrimination perception and alexithymia, rather than incorporating all three variables into a single integrated network. While this approach reduces network complexity and improves edge estimation stability, it precludes direct examination of the conditional association between shyness and alexithymia, as well as potential interaction effects among the three variables. To address this, we provide a three-community combined network as a sensitivity analysis in the **Supplementary Appendices 15–21**. Future research could construct integrated or longitudinal networks with larger samples to further validate and extend the present findings.

Third, sample representativeness limitations. At the regional level, the sample was drawn primarily from Sichuan and Fujian provinces. Differences in regional culture and economic development may introduce geographical bias and limit generalizability. Future studies should expand the sampling scope to include hearing-impaired college students from multiple regions and diverse backgrounds, and incorporate regional economic development level as a covariate. At the age level, the sample was restricted to hearing-impaired college students aged 18–22 years, making it difficult to generalize to other age groups (e.g., adolescents or middle-aged adults with hearing impairment). Subsequent research could broaden the age range to reveal age-related differences in psychological characteristics among hearing-impaired populations. At the group comparison level, this study did not include a hearing control group, making it unclear whether the observed network structure reflects characteristics specific to hearing-impaired college students or general psychological patterns among college students. Future research could add a hearing control group and conduct cross-group network comparisons to examine the moderating role of hearing impairment status in the association between discrimination perception and mental health.

Fourth, limitations of network analysis methodology. The BEI stability coefficients were 0.283 and 0.361—above the minimum threshold of 0.25 but below the preferred threshold of 0.50—requiring cautious interpretation of bridge nodes. Most cross-community edge weights were below 0.06, indicating small effect sizes with unverified clinical significance. These weak associations should not be interpreted as reflecting strong psychological mechanisms or causal pathways, and may be attributable to inherently modest relationships, omitted variables (e.g., social support, self-esteem), underestimation due to cross-sectional design, and measurement error. Replication in larger, more comprehensive studies is needed.

## Conclusion

This study used network analysis to explore the relationships between discrimination perception, shyness, and alexithymia in hearing-impaired college students. The findings suggest that discrimination perception nodes such as Pd2 (not being liked) and Pd5 (treated differently) may serve as potential targets for interventions addressing shyness and alexithymia in this population. Identifying these key and bridge symptoms could help create a better campus environment for hearing-impaired college students.

## Data Availability

The original contributions presented in the study are included in the article/**Supplementary Material**. Further inquiries can be directed to the corresponding author.

## Supplementary material

Supplementary Appendix 15 Network structure plot of the three-community combined network.

Supplementary Appendix 16 Stability test of bridge centrality nodes of the three-community combined network.

Supplementary Appendix 17 Edge accuracy of the three-community combined network (without vertical axis).

Supplementary Appendix 18 Edge accuracy of the three-community combined network (with vertical axis).

Supplementary Appendix 19 Edge difference tests of the three-community combined network.

Supplementary Appendix 20 Bridge centrality difference tests of the three-community combined network.

Supplementary Appendix 21 Bridge expected influence values of the three-community combined network.
