# Supplementary material for: Exploring the interplay of discrimination perception, alexithymia, and shyness among hearing-impaired college students: a network approach
Source: Front Psychiatry. 2026 Sep 24;17:1899912. doi: 10.3389/fpsyt.2026.1899912 (PMC13645756; doi:10.3389/fpsyt.2026.1899912)
Supplement: Supplementary file 1 [file DataSheet10.pdf]

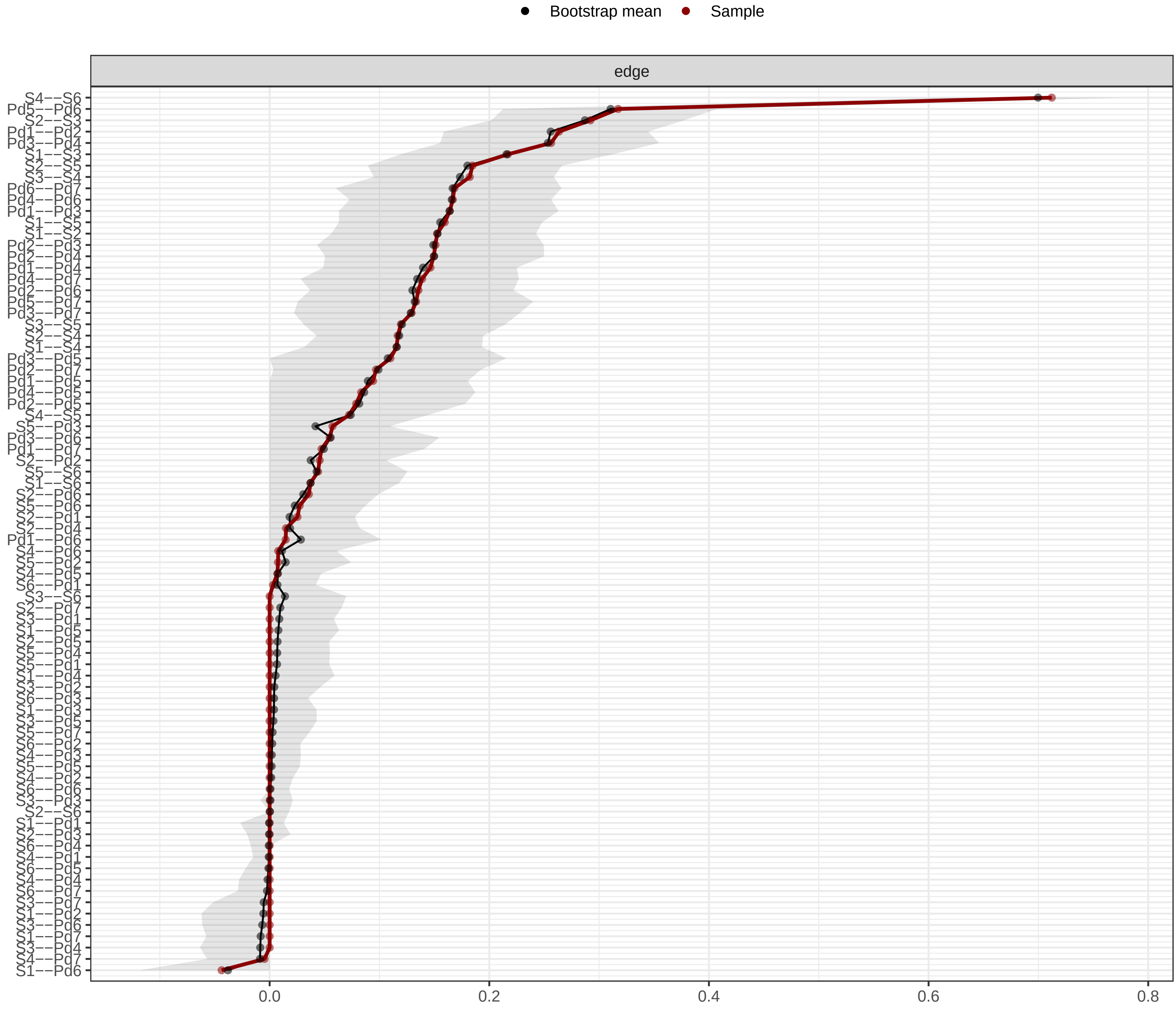

Bootstrap mean
Sample
edge
S4--S6
Pd5--Pd6
S2--S3
Pd1--Pd2
Pd3--Pd4
S1--S3
S2--S5
S3--S4
Pd6--Pd7
Pd4--Pd6
Pd1--Pd3
S1--S5
S1--S2
Pd2--Pd3
Pd2--Pd4
Pd1--Pd4
Pd4--Pd7
Pd2--Pd6
Pd5--Pd7
Pd3--Pd7
S3--S5
S2--S4
S1--S4
Pd3--Pd5
Pd2--Pd7
Pd1--Pd5
Pd4--Pd5
Pd2--Pd5
S4--S5
S5--Pd3
Pd3--Pd6
Pd1--Pd7
S2--Pd2
S5--S6
S1--S6
S2--Pd6
S5--Pd6
S2--Pd1
S2--Pd4
Pd1--Pd6
S4--Pd6
S5--Pd2
S4--Pd5
S6--Pd1
S3--S6
S2--Pd7
S3--Pd1
S1--Pd5
S2--Pd5
S5--Pd4
S5--Pd1
S1--Pd4
S3--Pd2
S6--Pd3
S1--Pd3
S3--Pd5
S5--Pd7
S6--Pd2
S4--Pd3
S5--Pd5
S4--Pd2
S6--Pd6
S3--Pd3
S2--S6
S1--Pd1
S2--Pd3
S6--Pd4
S4--Pd1
S6--Pd5
S4--Pd4
S6--Pd7
S3--Pd7
S1--Pd2
S3--Pd6
S1--Pd7
S3--Pd4
S4--Pd7
S1--Pd6
0.0
0.2
0.4
0.6
0.8
