# Supplementary material for: Exploring the interplay of discrimination perception, alexithymia, and shyness among hearing-impaired college students: a network approach
Source: Front Psychiatry. 2026 Sep 24;17:1899912. doi: 10.3389/fpsyt.2026.1899912 (PMC13645756; doi:10.3389/fpsyt.2026.1899912)
Supplement: Supplementary file 2 [file DataSheet11.pdf]

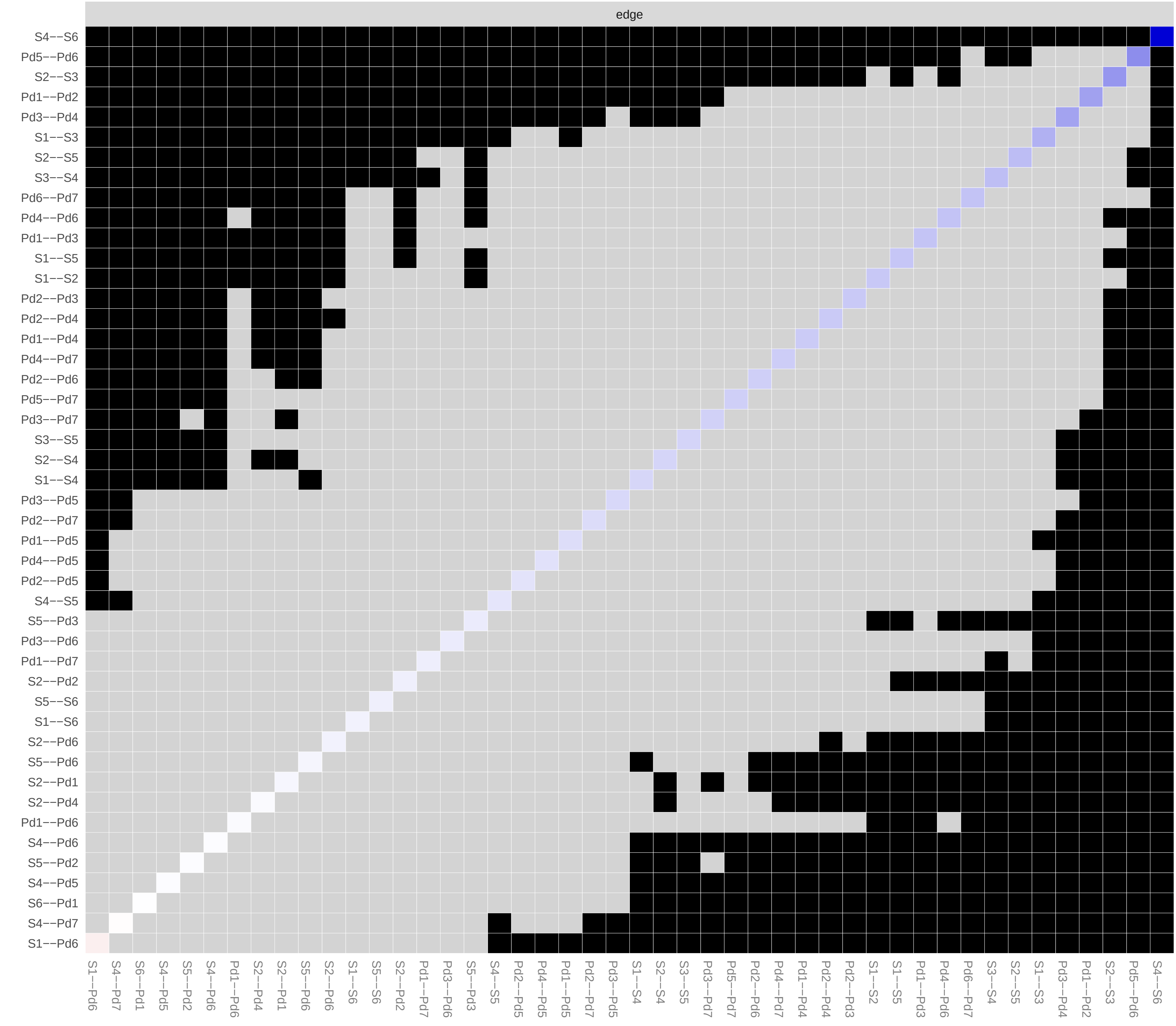
edge
S4−−S6
Pd5−−Pd6
S2−−S3
Pd1−−Pd2
Pd3−−Pd4
S1−−S3
S2−−S5
S3−−S4
Pd6−−Pd7
Pd4−−Pd6
Pd1−−Pd3
S1−−S5
S1−−S2
Pd2−−Pd3
Pd2−−Pd4
Pd1−−Pd4
Pd4−−Pd7
Pd2−−Pd6
Pd5−−Pd7
Pd3−−Pd7
S3−−S5
S2−−S4
S1−−S4
Pd3−−Pd5
Pd2−−Pd7
Pd1−−Pd5
Pd4−−Pd5
Pd2−−Pd5
S4−−S5
S5−−Pd3
Pd3−−Pd6
Pd1−−Pd7
S2−−Pd2
S5−−S6
S1−−S6
S2−−Pd6
S5−−Pd6
S2−−Pd1
S2−−Pd4
Pd1−−Pd6
S4−−Pd6
S5−−Pd2
S4−−Pd5
S6−−Pd1
S4−−Pd7
S1−−Pd6
