# Supplementary material for: Exploring the interplay of discrimination perception, alexithymia, and shyness among hearing-impaired college students: a network approach
Source: Front Psychiatry. 2026 Sep 24;17:1899912. doi: 10.3389/fpsyt.2026.1899912 (PMC13645756; doi:10.3389/fpsyt.2026.1899912)
Supplement: Supplementary file 3 [file DataSheet12.pdf]

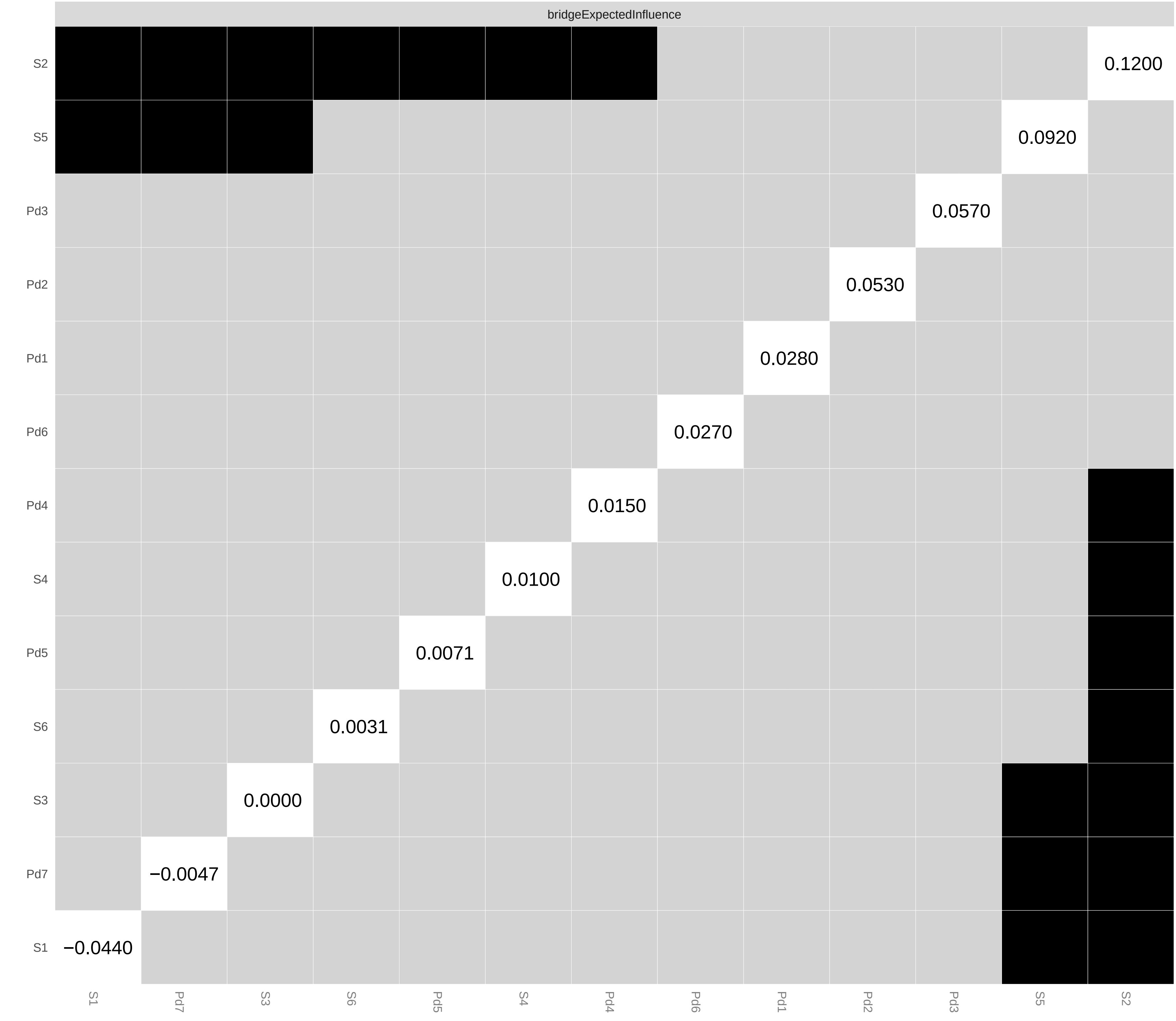
bridgeExpectedInfluence
S2
S5
Pd3
Pd2
Pd1
Pd6
Pd4
S4
Pd5
S6
S3
Pd7
S1
0.1200
0.0920
0.0570
0.0530
0.0280
0.0270
0.0150
0.0100
0.0071
0.0031
0.0000
−0.0047
−0.0440
S1
Pd7
S3
S6
Pd5
S4
Pd4
Pd6
Pd1
Pd2
Pd3
S5
S2
