# Supplementary material for: Exploring the interplay of discrimination perception, alexithymia, and shyness among hearing-impaired college students: a network approach
Source: Front Psychiatry. 2026 Sep 24;17:1899912. doi: 10.3389/fpsyt.2026.1899912 (PMC13645756; doi:10.3389/fpsyt.2026.1899912)
Supplement: Supplementary file 5 [file DataSheet14.pdf]

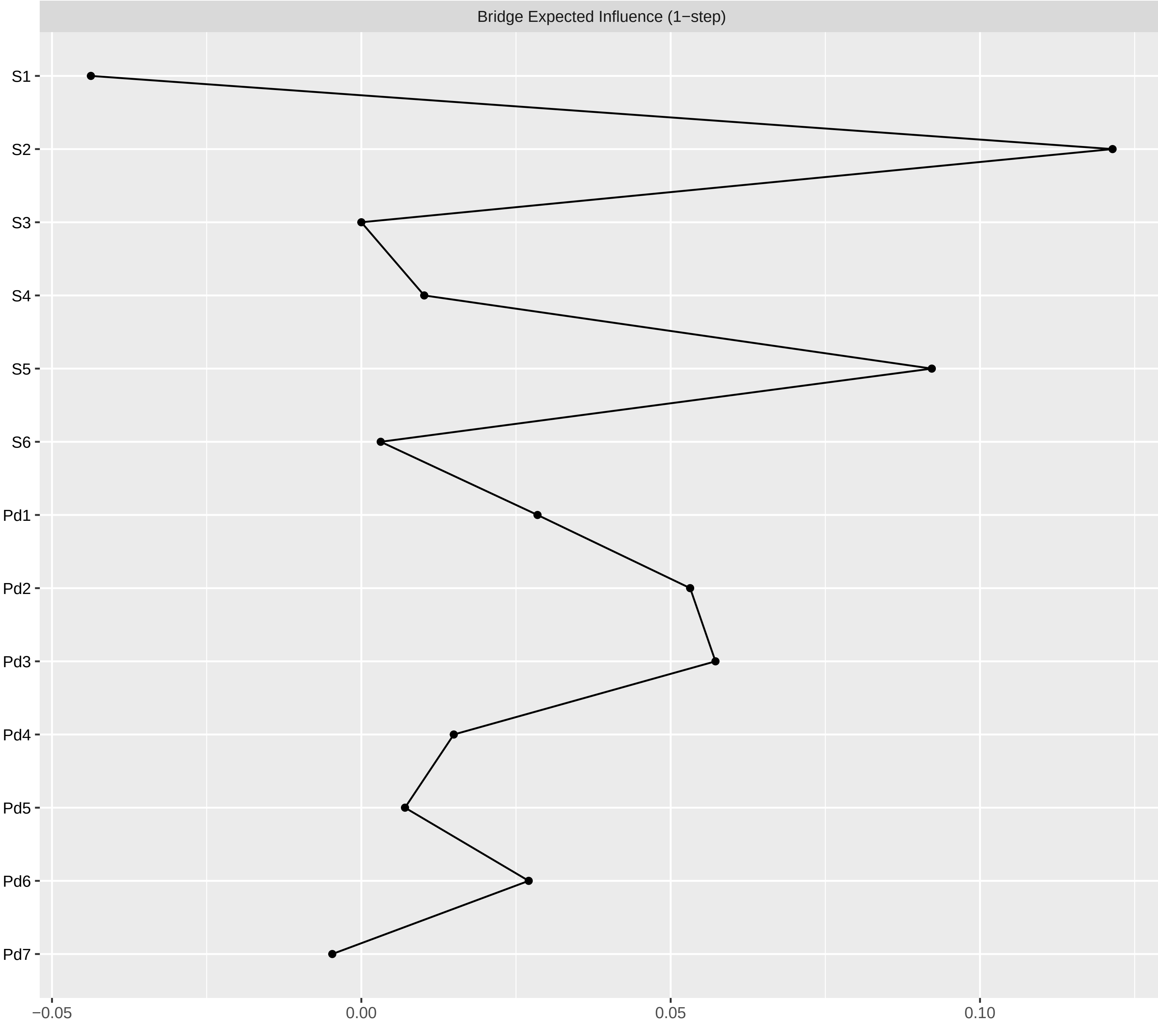
Bridge Expected Influence (1−step)
S1
S2
S3
S4
S5
S6
Pd1
Pd2
Pd3
Pd4
Pd5
Pd6
Pd7
−0.05
0.00
0.05
0.10
