# Supplementary material for: Exploring the interplay of discrimination perception, alexithymia, and shyness among hearing-impaired college students: a network approach
Source: Front Psychiatry. 2026 Sep 24;17:1899912. doi: 10.3389/fpsyt.2026.1899912 (PMC13645756; doi:10.3389/fpsyt.2026.1899912)
Supplement: Supplementary file 8 [file DataSheet4.pdf]

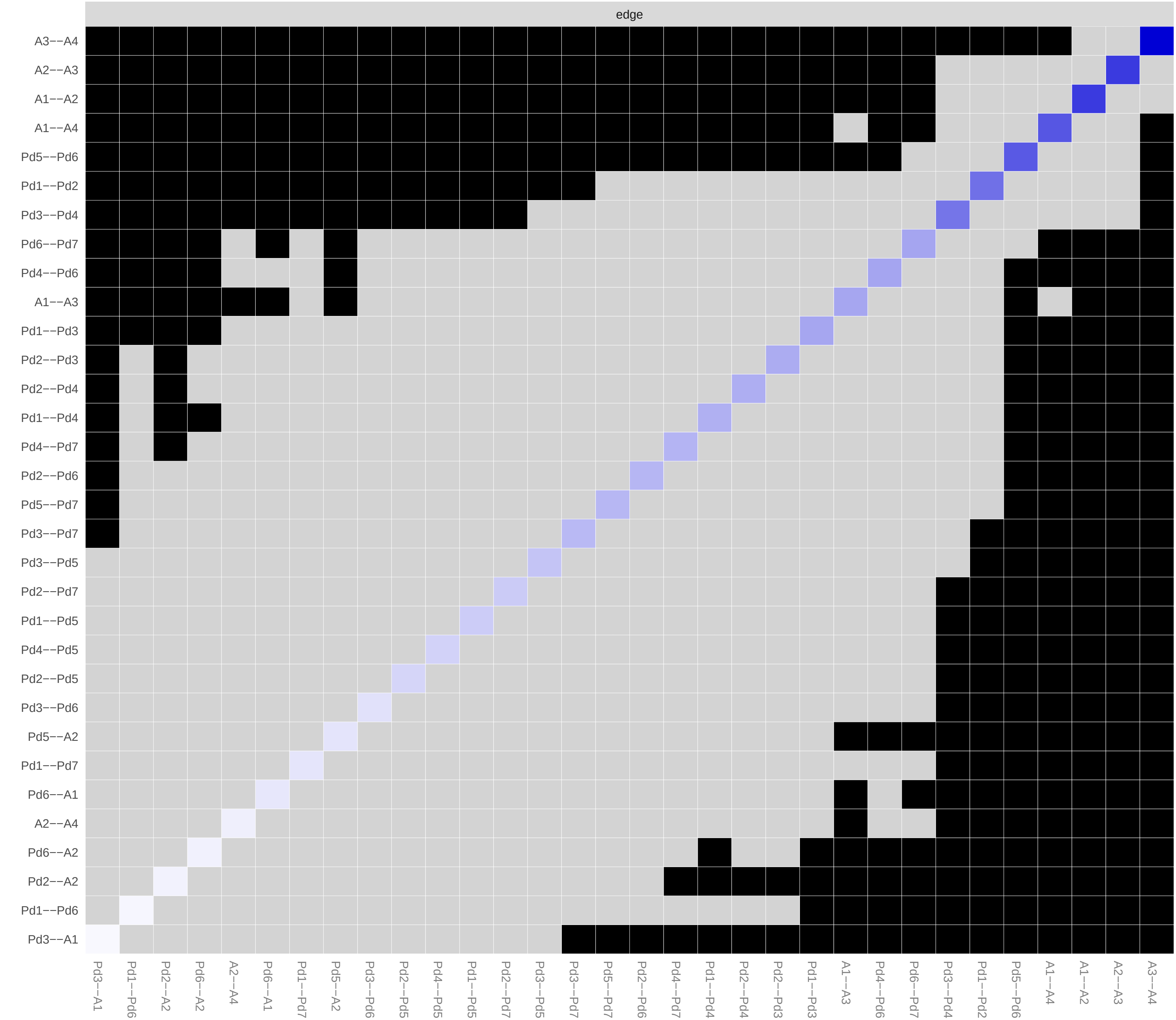

edge
A3--A4
A2--A3
A1--A2
A1--A4
Pd5--Pd6
Pd1--Pd2
Pd3--Pd4
Pd6--Pd7
Pd4--Pd6
A1--A3
Pd1--Pd3
Pd2--Pd3
Pd2--Pd4
Pd1--Pd4
Pd4--Pd7
Pd2--Pd6
Pd5--Pd7
Pd3--Pd7
Pd3--Pd5
Pd2--Pd7
Pd1--Pd5
Pd4--Pd5
Pd2--Pd5
Pd3--Pd6
Pd5--A2
Pd1--Pd7
Pd6--A1
A2--A4
Pd6--A2
Pd2--A2
Pd1--Pd6
Pd3--A1
Pd3--A1
Pd1--Pd6
Pd2--A2
Pd6--A2
A2--A4
Pd6--A1
Pd1--Pd7
Pd5--A2
Pd3--Pd6
Pd2--Pd5
Pd4--Pd5
Pd1--Pd5
Pd2--Pd7
Pd3--Pd5
Pd3--Pd7
Pd5--Pd7
Pd2--Pd6
Pd4--Pd7
Pd1--Pd4
Pd2--Pd4
Pd2--Pd3
Pd1--Pd3
A1--A3
Pd4--Pd6
Pd6--Pd7
Pd3--Pd4
Pd1--Pd2
Pd5--Pd6
A1--A4
A1--A2
A2--A3
A3--A4
