# Supplementary material for: Exploring the interplay of discrimination perception, alexithymia, and shyness among hearing-impaired college students: a network approach
Source: Front Psychiatry. 2026 Sep 24;17:1899912. doi: 10.3389/fpsyt.2026.1899912 (PMC13645756; doi:10.3389/fpsyt.2026.1899912)
Supplement: Supplementary file 9 [file DataSheet5.pdf]

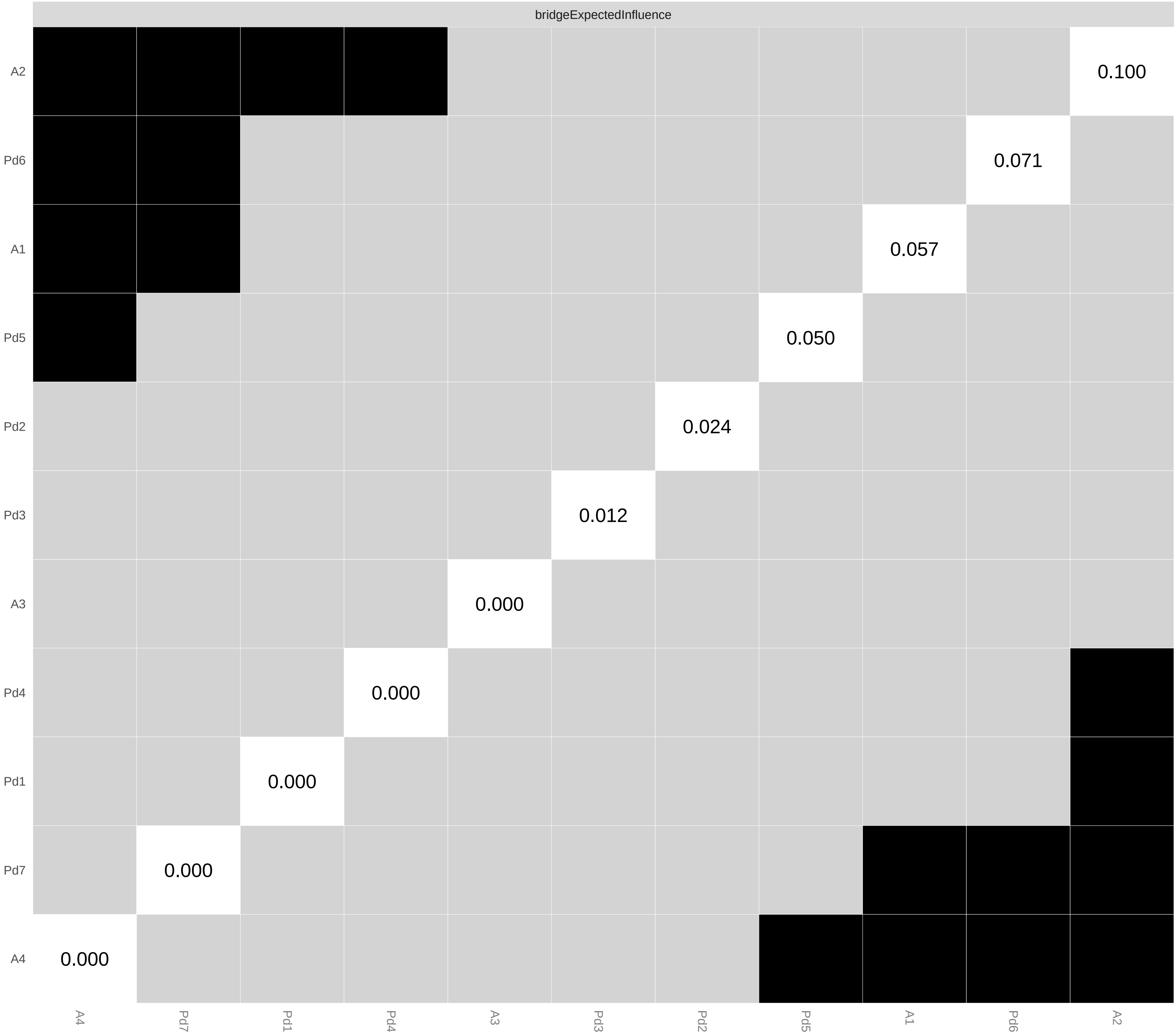
bridgeExpectedInfluence
A2
Pd6
A1
Pd5
Pd2
Pd3
A3
Pd4
Pd1
Pd7
A4
0.100
0.071
0.057
0.050
0.024
0.012
0.000
0.000
0.000
0.000
0.000
A4
Pd7
Pd1
Pd4
A3
Pd3
Pd2
Pd5
A1
Pd6
A2
