# Supplementary material for: Exploring the interplay of discrimination perception, alexithymia, and shyness among hearing-impaired college students: a network approach
Source: Front Psychiatry. 2026 Sep 24;17:1899912. doi: 10.3389/fpsyt.2026.1899912 (PMC13645756; doi:10.3389/fpsyt.2026.1899912)
Supplement: Supplementary file 10 [file DataSheet6.pdf]

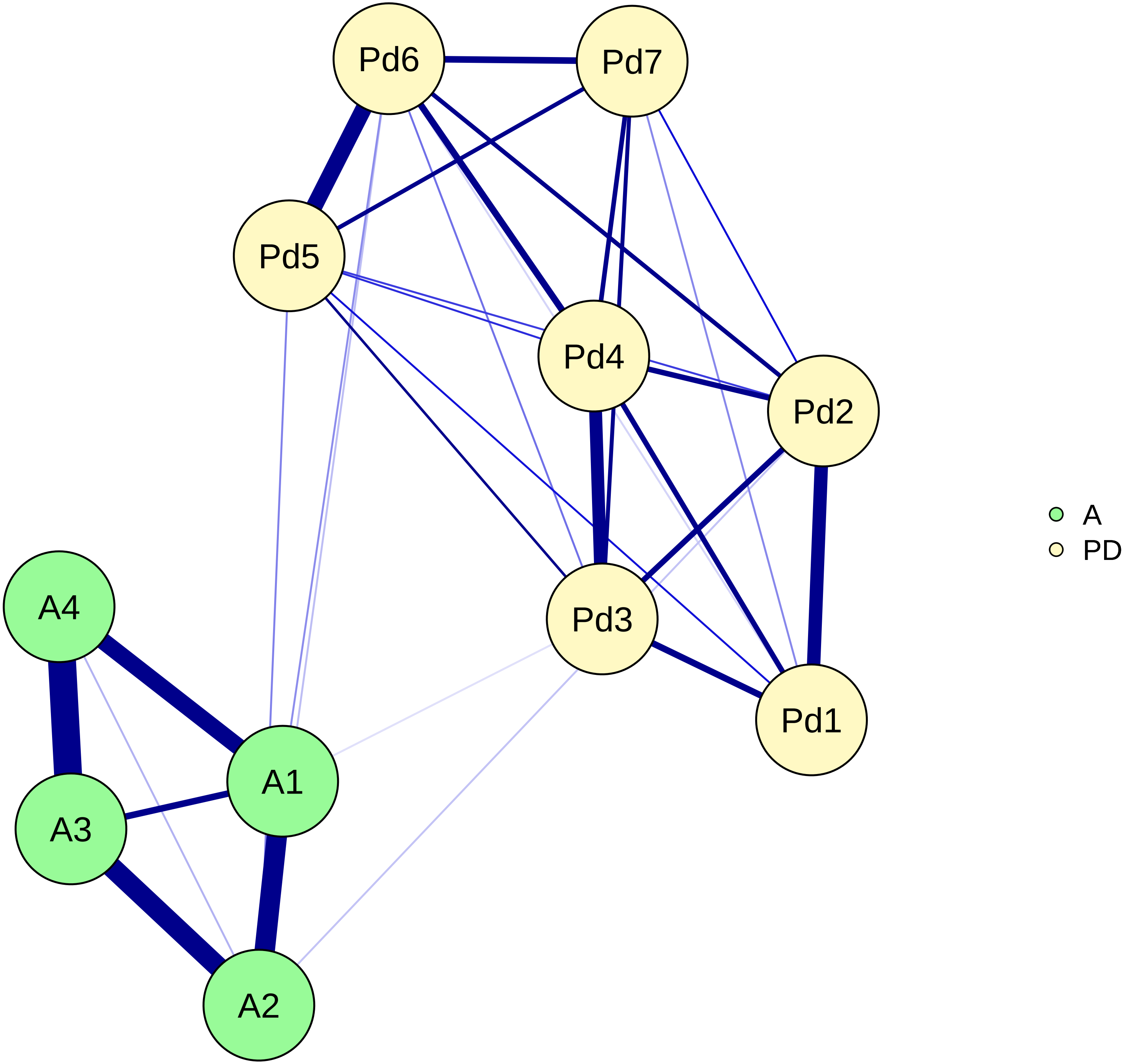
Pd6
Pd7
Pd5
Pd4
Pd2
Pd3
Pd1
A4
A1
A3
A2
A
PD
