# Supplementary material for: Exploring the interplay of discrimination perception, alexithymia, and shyness among hearing-impaired college students: a network approach
Source: Front Psychiatry. 2026 Sep 24;17:1899912. doi: 10.3389/fpsyt.2026.1899912 (PMC13645756; doi:10.3389/fpsyt.2026.1899912)
Supplement: Supplementary file 11 [file DataSheet7.pdf]

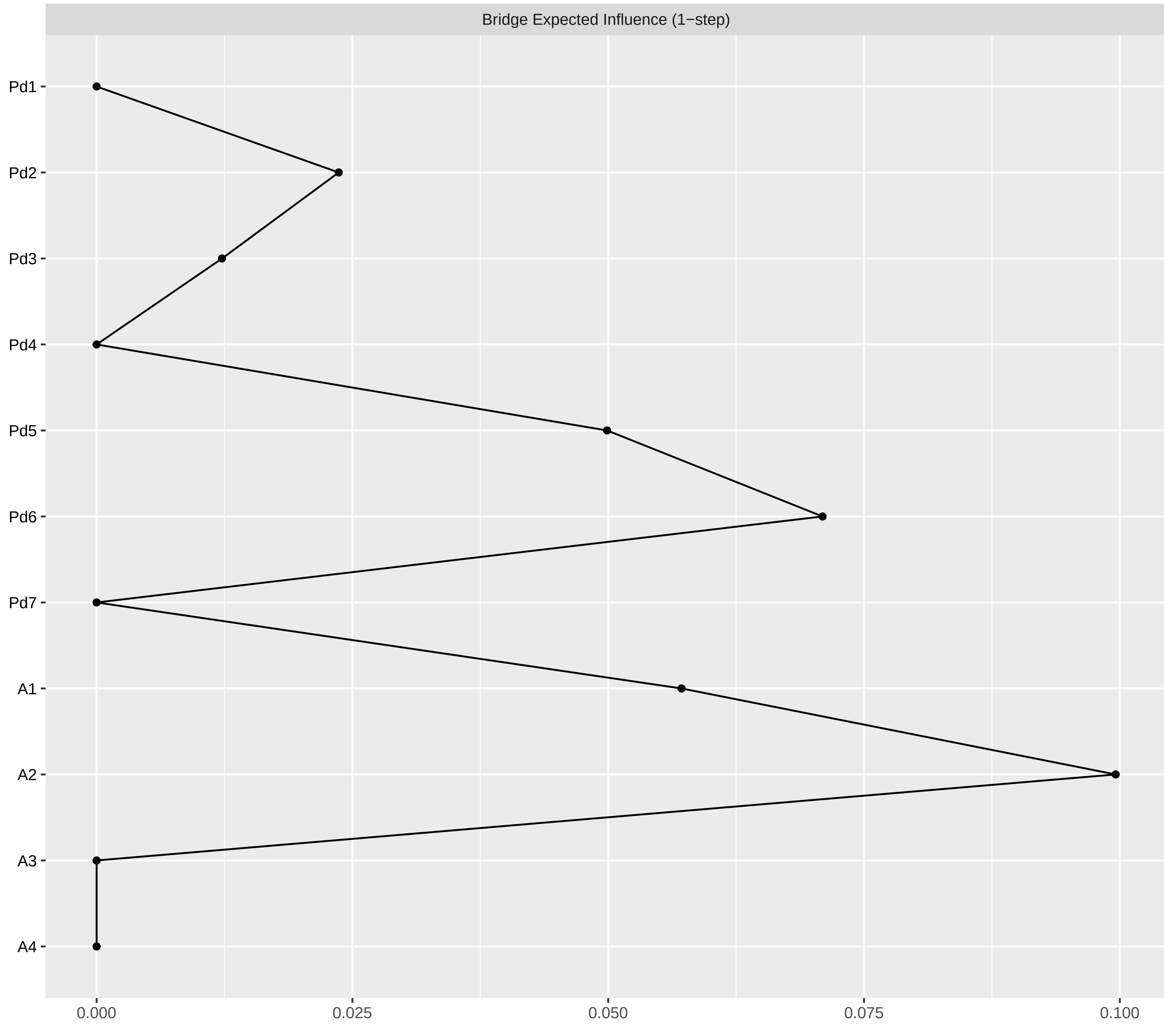

Bridge Expected Influence (1−step)
Pd1
Pd2
Pd3
Pd4
Pd5
Pd6
Pd7
A1
A2
A3
A4
0.000
0.025
0.050
0.075
0.100
