# Supplementary material for: Exploring the interplay of discrimination perception, alexithymia, and shyness among hearing-impaired college students: a network approach
Source: Front Psychiatry. 2026 Sep 24;17:1899912. doi: 10.3389/fpsyt.2026.1899912 (PMC13645756; doi:10.3389/fpsyt.2026.1899912)
Supplement: Supplementary file 12 [file DataSheet9.pdf]

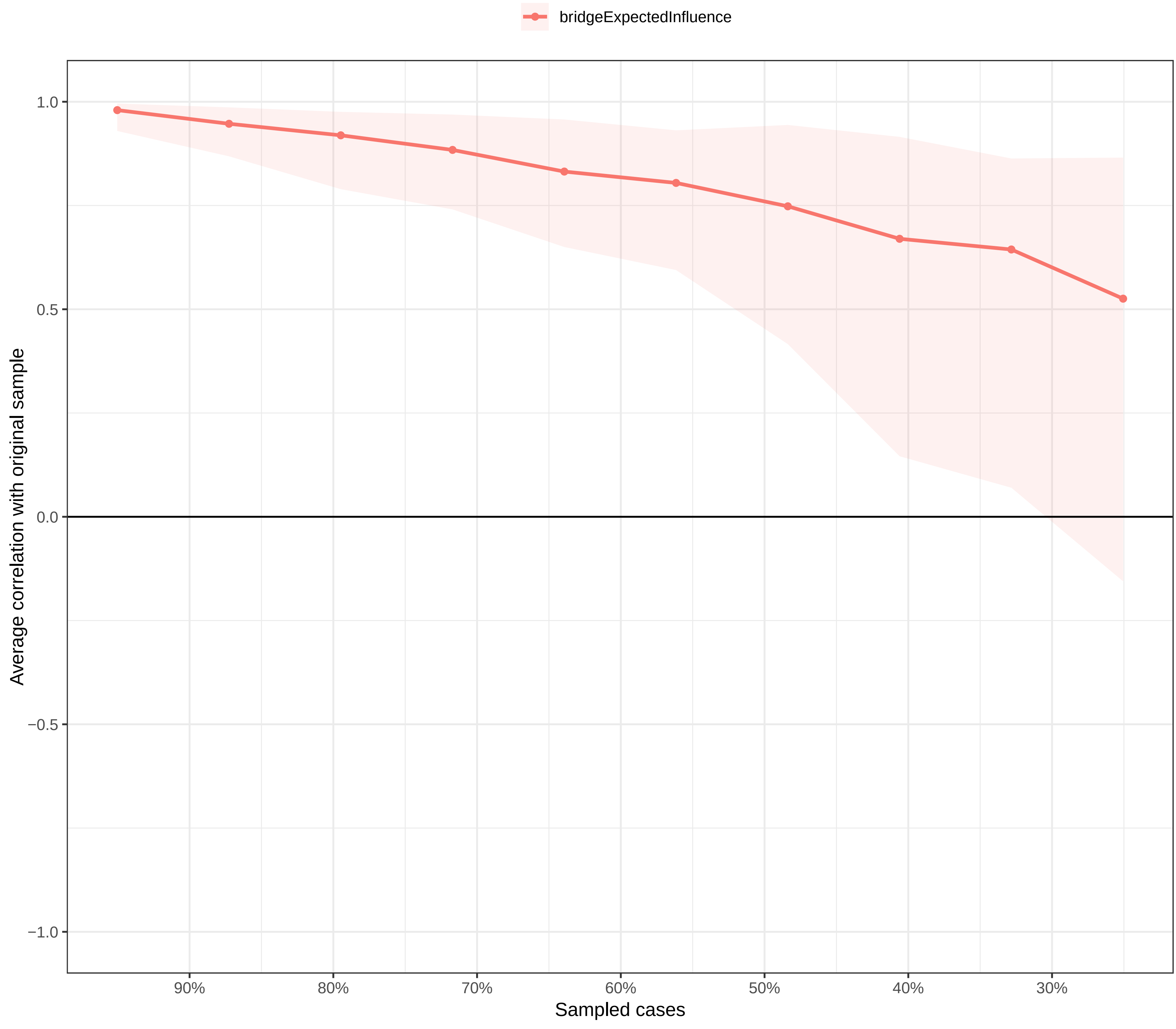
bridgeExpectedInfluence
1.0
0.5
0.0
−0.5
−1.0
Average correlation with original sample
90%
80%
70%
60%
50%
40%
30%
Sampled cases
