# Supplementary material for: Exploring the interplay of discrimination perception, alexithymia, and shyness among hearing-impaired college students: a network approach
Source: Front Psychiatry. 2026 Sep 24;17:1899912. doi: 10.3389/fpsyt.2026.1899912 (PMC13645756; doi:10.3389/fpsyt.2026.1899912)
Supplement: Supplementary file 13 [file SupplementaryFile1.pdf]

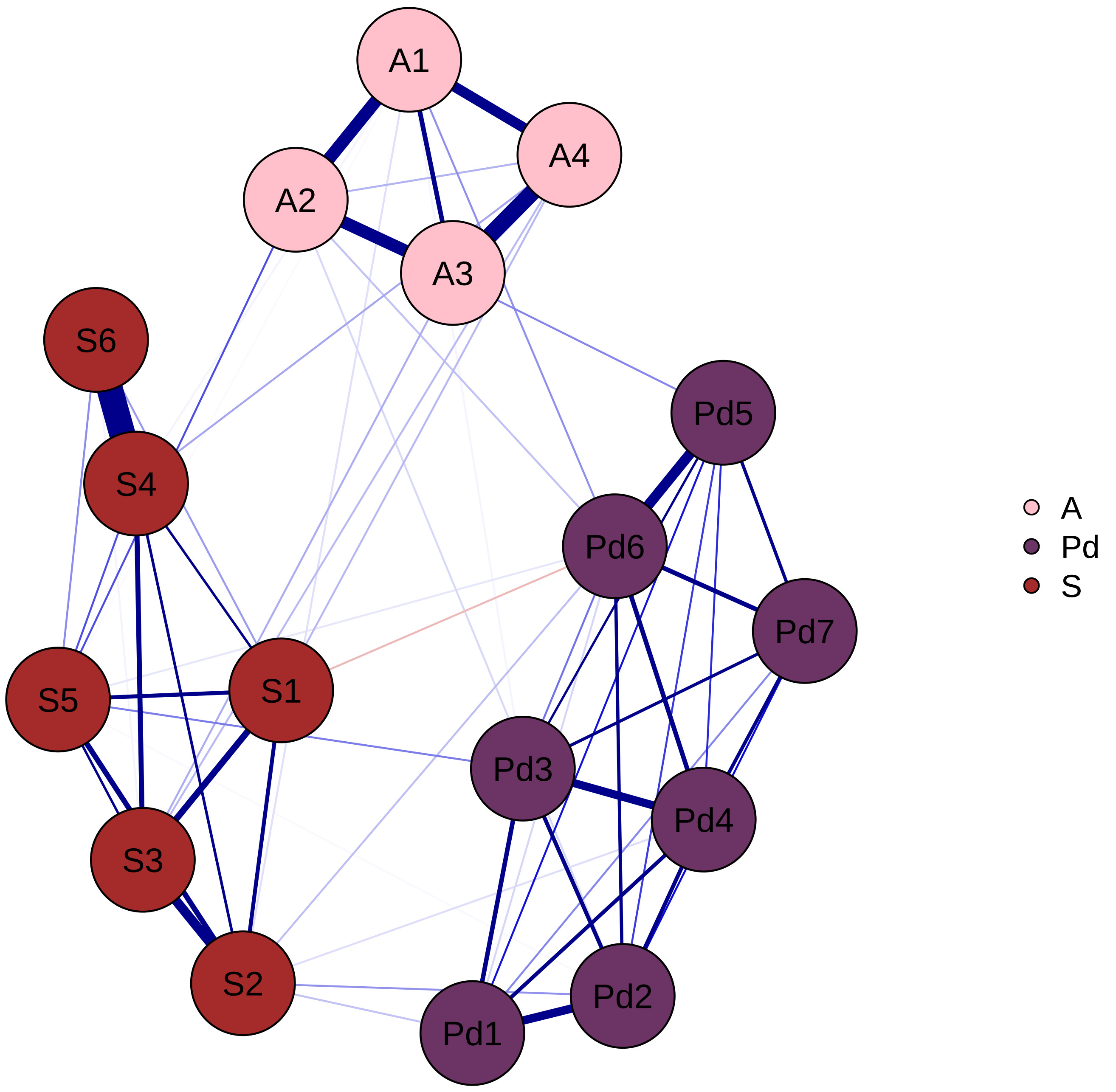
A1
A2
A3
A4
S6
S4
S5
S1
S3
S2
Pd5
Pd6
Pd7
Pd3
Pd4
Pd2
Pd1
A
Pd
S
