# Supplementary material for: Exploring the interplay of discrimination perception, alexithymia, and shyness among hearing-impaired college students: a network approach
Source: Front Psychiatry. 2026 Sep 24;17:1899912. doi: 10.3389/fpsyt.2026.1899912 (PMC13645756; doi:10.3389/fpsyt.2026.1899912)
Supplement: Supplementary file 16 [file SupplementaryFile4.pdf]

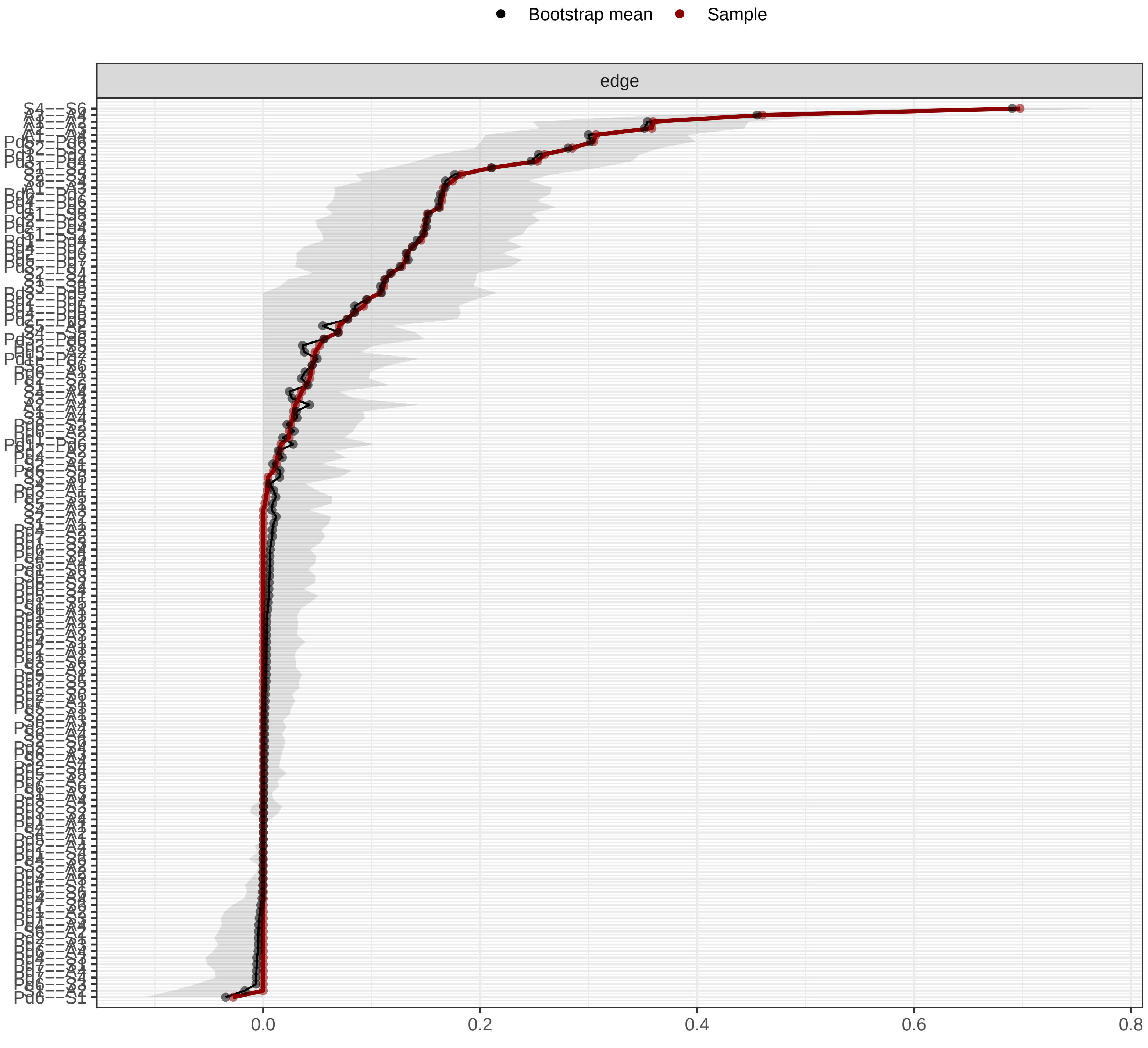
Bootstrap mean
Sample
edge
S4--S6
Pd6--S1
0.0
0.2
0.4
0.6
0.8
