# Supplementary material for: Exploring the interplay of discrimination perception, alexithymia, and shyness among hearing-impaired college students: a network approach
Source: Front Psychiatry. 2026 Sep 24;17:1899912. doi: 10.3389/fpsyt.2026.1899912 (PMC13645756; doi:10.3389/fpsyt.2026.1899912)
Supplement: Supplementary file 17 [file SupplementaryFile5.pdf]

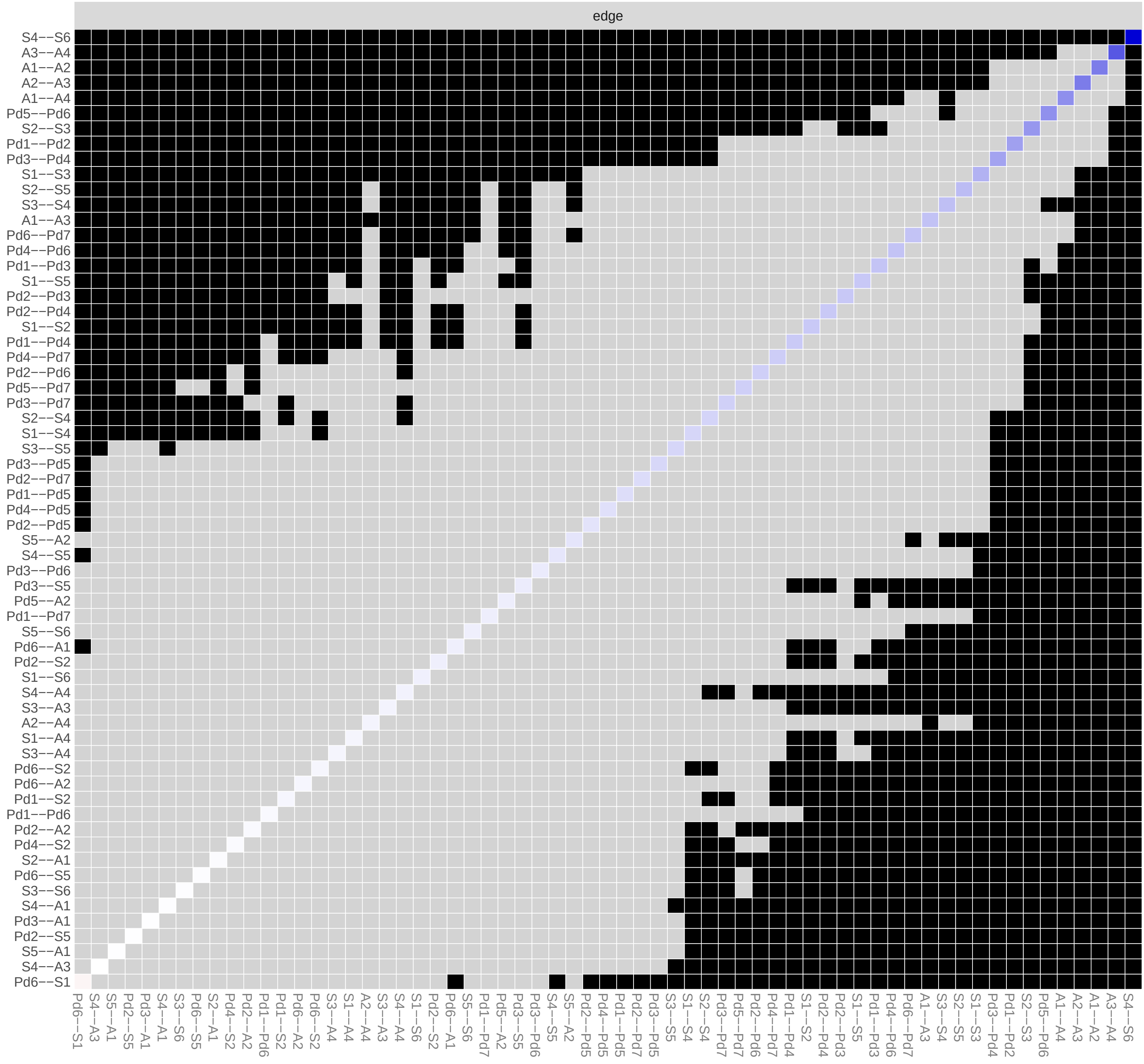

edge
S4--S6
A3--A4
A1--A2
A2--A3
A1--A4
Pd5--Pd6
S2--S3
Pd1--Pd2
Pd3--Pd4
S1--S3
S2--S5
S3--S4
A1--A3
Pd6--Pd7
Pd4--Pd6
Pd1--Pd3
S1--S5
Pd2--Pd3
Pd2--Pd4
S1--S2
Pd1--Pd4
Pd4--Pd7
Pd2--Pd6
Pd5--Pd7
Pd3--Pd7
S2--S4
S1--S4
S3--S5
Pd3--Pd5
Pd2--Pd7
Pd1--Pd5
Pd4--Pd5
Pd2--Pd5
S5--A2
S4--S5
Pd3--Pd6
Pd3--S5
Pd5--A2
Pd1--Pd7
S5--S6
Pd6--A1
Pd2--S2
S1--S6
S4--A4
S3--A3
A2--A4
S1--A4
S3--A4
Pd6--S2
Pd6--A2
Pd1--S2
Pd1--Pd6
Pd2--A2
Pd4--S2
S2--A1
Pd6--S5
S3--S6
S4--A1
Pd3--A1
Pd2--S5
S5--A1
S4--A3
Pd6--S1
Pd6--S1
S4--A3
S5--A1
Pd2--S5
Pd3--A1
S4--A1
S3--S6
Pd6--S5
S2--A1
Pd4--S2
Pd2--A2
Pd1--Pd6
Pd1--S2
Pd6--A2
Pd6--S2
S3--A4
S1--A4
A2--A4
S3--A3
S4--A4
S1--S6
Pd2--S2
Pd6--A1
S5--S6
Pd1--Pd7
Pd5--A2
Pd3--S5
Pd3--Pd6
S4--S5
S5--A2
Pd2--Pd5
Pd4--Pd5
Pd1--Pd5
Pd2--Pd7
Pd3--Pd5
S3--S5
S1--S4
S2--S4
Pd3--Pd7
Pd5--Pd7
Pd2--Pd6
Pd4--Pd7
Pd1--Pd4
S1--S2
Pd2--Pd4
Pd2--Pd3
S1--S5
Pd1--Pd3
Pd4--Pd6
Pd6--Pd7
A1--A3
S3--S4
S2--S5
S1--S3
Pd3--Pd4
Pd1--Pd2
S2--S3
Pd5--Pd6
A1--A4
A2--A3
A1--A2
A3--A4
S4--S6
