# Supplementary material for: Exploring the interplay of discrimination perception, alexithymia, and shyness among hearing-impaired college students: a network approach
Source: Front Psychiatry. 2026 Sep 24;17:1899912. doi: 10.3389/fpsyt.2026.1899912 (PMC13645756; doi:10.3389/fpsyt.2026.1899912)
Supplement: Supplementary file 18 [file SupplementaryFile6.pdf]

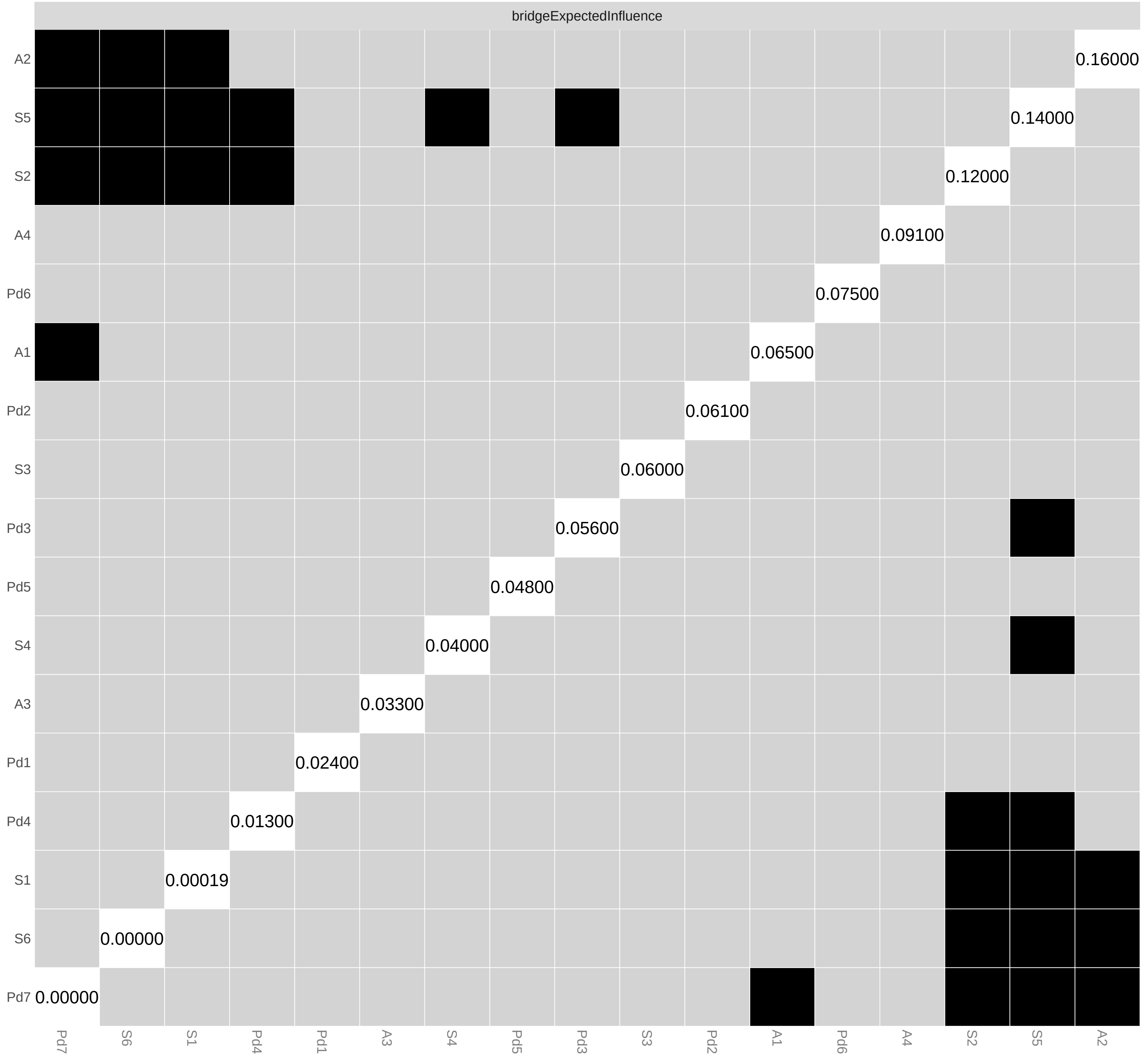
bridgeExpectedInfluence
A2
S5
S2
A4
Pd6
A1
Pd2
S3
Pd3
Pd5
S4
A3
Pd1
Pd4
S1
S6
Pd7
0.16000
0.14000
0.12000
0.09100
0.07500
0.06500
0.06100
0.06000
0.05600
0.04800
0.04000
0.03300
0.02400
0.01300
0.00019
0.00000
0.00000
Pd7
S6
S1
Pd4
Pd1
A3
S4
Pd5
Pd3
S3
Pd2
A1
Pd6
A4
S2
S5
A2
